# The dynamics of humoral immune responses following SARS-CoV-2 infection and the potential for reinfection

**DOI:** 10.1099/jgv.0.001439

**Published:** 2020-05-20

**Authors:** Paul Kellam, Wendy Barclay

**Affiliations:** ^1^​ Department of Infectious Diseases, Faulty of Medicine, Imperial College London, London, W2 1NY, UK; ^2^​ Kymab Ltd, The Bennet Building (B930), Babraham Research Campus, Cambridge, CB22 3AT, UK

**Keywords:** SARS-CoV-2, COVID-19, serology, reinfection, antibodies

## Abstract

SARS-CoV-2 is a novel coronavirus that is the causative agent of coronavirus infectious disease 2019 (COVID-19). As of 17 April 2020, it has infected 2 114 269 people, resulting in 145 144 deaths. The timing, magnitude and longevity of humoral immunity is not yet understood for SARS-CoV-2. Nevertheless, understanding this is urgently required to inform the likely future dynamics of the pandemic, to guide strategies to allow relaxation of social distancing measures and to understand how to deploy limiting vaccine doses when they become available to achieve maximum impact. SARS-CoV-2 is the seventh human coronavirus to be described. Four human coronaviruses circulate seasonally and cause common colds. Two other coronaviruses, SARS and MERS, have crossed from animal sources into humans but have not become endemic. Here we review what is known about the human humoral immune response to epidemic SARS CoV and MERS CoV and to the seasonal, endemic coronaviruses. Then we summarize recent, mostly non-peer reviewed, studies into SARS-CoV-2 serology and reinfection in humans and non-human primates and summarize current pressing research needs.

## Serological decline after MERS CoV and SARS CoV infection

A few studies have assessed antibody titres to MERS CoV and SARS CoV in the months and years following primary infection. Robust immune responses with long-lived (>1 year) functional antibodies were seen following severe MERS CoV infections or in those people with prolonged virus shedding [1] [2]. This was also observed in a small study of MERS CoV infections, where neutralizing antibodies were detectable in six (86 %) out of seven persons who had previously had severe MERS (including five with pneumonia) for at least 34 months after infection. However, in this small group there was evidence of antibody waning; 4/7 showed 4- to 16-fold reduction in nucleocapsid-binding titres and 4/7 show a twofold reduction in neutralizing titres over 34 months, with 4/7 assessed as having a low neutralizing titres throughout [3]. After mild or asymptomatic MERS CoV infections, antibody responses were either limited or rapidly declined. Although the numbers are small, no neutralizing antibody response was seen in 4/6 [1]) and 3/6 ([4] mild MERS CoV infections for some, not even immediately after infection [1]. In a separate study of 280 contacts of 26 confirmed MERS CoV index cases, 12 contacts likely to have been infected were identified. Seven out of 12 contacts sampled within 4–14 days of index contact were virus genome-positive by RT-PCR but serologically negative (actively infected), whereas 5/7 were virus genome-negative, but had detectable binding and neutralizing antibody titres (infected and recovered) [5].

Similarly, although SARS CoV was largely associated with symptomatic disease, antibodies decline over time. In a 3-year follow-up of hospitalized SARS CoV patients, SARS CoV IgG-binding titres were undetectable in 19.4 % of people by 30 months post-infection and neutralizing titres were undetectable in 11.1 % of people at this time [6]. Consistent with this observation, a study of 98 SARS patients over 2 years showed that all had detectable antibody binding titres over 2 years, but that, in a subset, titres declined over this period. Eighteen individuals with neutralizing antibodies had titres that peaked on day 30 and then decayed gradually so that by 2 years 1/18 had no detectable neutralizing antibodies, and the remaining patients had low antibody titres close to background levels [7]. Similarly, in a study following 176 previously SARS CoV-infected people, the enzyme-linked immunosorbent assay (ELISA) optical densities (ODs) that indicate antibody titre reduced by 33 % within 1 year, 46 % by 2 years and ~75 % by 3 years [8]. Nevertheless, other long-term follow-up studies of SARS CoV showed that although antibody titres decline over over 2 [9] and 3 years [10], neutralizing activity was present in 89 % (17/19) of the recovered patients at 36 months, although the ability of sera to neutralize virus declined from 96 % inhibition at month 3 to 48 % at month 36 [10]. Although antibody titres to SARS CoV can be detected in people 12 years after infection, over 70 % the people studied (*n*=20) had extremely low titres [11], and so at 3 and 12 years post-infection SARS CoV antibody titres are likely to be very limited for virus neutralization, with little or no ability to protect a person from reinfection. However, this requires experimental determination.

Although limited in size, studies of MERS and SARS CoV indicate that total binding antibodies and neutralizing antibodies decrease to a level where by 2–3 years everyone previously infected will have minimal detectable antibody response, but those suffering more severe disease have the highest titre antibody responses for longer. Although the time-dependent decay of neutralizing antibody titres implies a lack of protection from reinfection by MERS and SARS CoV, this cannot be concluded unequivocally, due to lack of epidemic spread allowing reinfection. It is, however, suggestive of the potential for a population-level reduction in protection from reinfection by epidemic CoVs over a short period of time, dependent in some on initial disease severity.

## Seroconversion rates to seasonal human coronaviruses

One indication of the strength of immune protection from coronavirus infection is to consider what is known for the endemic seasonal CoVs, namely the genetically related alphacoronaviruses, NL63 and 229E, and the genetically related betacoronaviruses, HKU1 and OC43. There is some evidence for antigenic cross-protection between the human CoVs in the same genetic group. A cross-sectional seroprevalence study for seasonal human alphacoronaviruses NL63 or 229E showed that 75 and 65 % of children in the age group 2.5–3.5 years are seropositive for NL63 and 229E, respectively, and most children are seropositive by 6 years [12]. In adults, respiratory infection by human seasonal CoVs accounted for 22 % (43/195) [13] and 25 % [14] of acute respiratory illness. Therefore, the ability of human seasonal coronaviruses to infect adults who have likely been infected as children can be accounted for by either virus escape from neutralization (drift), reinfection with a heterologous CoV of a different genotype (alpha- followed by betacoronavirus infections, or vice versa) due to lack of cross-protective antibodies, or reinfection with homologous coronavirus due to sub-protective or waning antibody responses.

The lack of extensive time-resolved virus genetic data linked to serology studies of extant and historic strains of the four seasonal human coronaviruses makes the contribution of virus genetic drift to escape from pre-existing protective immune response difficult to judge. The evolutionary genetics of coronaviruses, especially in animals, however, shows considerable genetic diversity within coronavirus species, largely driven by high rates of substitution and recombination. For infectious bronchitis virus (IBV) of chickens, the existence of many serotypes, with little cross-protective immunity between them, is attributed to both small and large numbers of amino acids substitutions in the spike gene, supporting the view that immune escape through genetic drift in animal coronavirus is common [15]. Work on the human endemic coronavirus OC43 suggests that genetic drift is similarly important, with considerable genetic diversity in the spike gene suggesting circulation of distinct OC43 variants [16]. Further, genetic drift mapping to sugar-binding domains in S protein of CoV OC43 suggests that drift may contribute to persistence of this genotype in the human population [17]. Similar studies on other endemic CoV genotypes are lacking and whether the use of different cell receptors constrains genetic variation in the spike gene of some human coronavirus more than others is not known, but infection due to immune escape through genetic drift seems important for coronaviruses. Waning of the neutralizing antibody response also seems to contribute to coronavirus reinfection. Whether coronaviruses encode specific proteins whose action is to limit the adaptive immune response or the spike protein is poor at initiating long-lived plasma cells is not known, but potential consequences of declining humoral immunity can be observed.

## Reinfection by seasonal human coronaviruses in the community

A small number of studies have attempted to detect reinfection by endemic CoVs in the community. In a cohort study of community-acquired and childhood pneumonia admissions to hospital in Kenya, reinfections by human coronavirus NL63 were detected over a 6-month period (December–May 2010) in 46 out of 163 patients (28 %) [18]. Most reinfections resulted in low virus titres and decreased disease. However, for a small number (11 %), reinfection resulted in higher virus shedding compared to the previous infection, with the caveat that the peak viral genome load in the first infection could have been missed in the sampling window. When reinfections occurred up to 80 days after first infection, the secondary infection virus load was usually low. However, reinfection after 80 days could result in high viral genome load, compatible with such viruses being capable of onward transmission. Sequence analysis of paired viral samples from the same individual reinfected after 80 days suggested reinfection was by a homologous CoV [18], although no antibody levels were measured in this study.

In a recent population study from the FLUWATCH project, over 5 seasons (2006–7 to 2010–11) the seasonal CoVs NL63, 229E and OC43 were detected at a rate of 390 infections [95 % confidence interval (CI): 338–448] per 100 000 person weeks. The rates of infection stratified by age showed a bimodal distribution with peaks at ages 0–4 and 16–44, consistent with previous serology studies. Importantly, eight subjects had more than one consecutive coronavirus infection. Of these, no participants had the same coronaviruses strain twice, with modelling suggesting that this provides some evidence for lasting immunity. Nonetheless, analysis of the CoV infection pairs per person shows these small numbers partition into 4/8 having a reinfection within 7–15 weeks, whereas 4/8 have a reinfection between 23–56 weeks. The former group all comprise infection–reinfection with heterologous alpha- (NL63 or 229E) and beta- (OC43) CoVs pairs, consistent with lack of serological cross-protection, whereas 3/8 of the latter group had homologous reinfection of alphacoronaviruses [19]. Although too small in numbers to be definitive, this suggests that serological protection from reinfection does exist but that it declines over a year, when infection with a virus of the same genotype becomes possible.

Evidence to support seroprotection against homologous virus genotypes exists in children, using serology assays specific for the carboxyl-terminal region of the nucleocapsid protein of each of the four viruses. Seroconversion to NL63 (alphacoronavirus) and OC43 (betacoronavirus) occurs more frequently in children in both households and in hospitals. When examining small numbers of reinfections, seroconversion to NL63 was correlated with protection from infection by 229E (both alphacoronaviruses). Similarly, seroconversion to OC43 can protect from reinfection by HKU1 (both betacoronaviruses). However, reciprocal protection (229E protects against NL63 and HKU1 against OC43) did not occur [20], suggesting that even homologous protection by genetically related CoV is not immunologically simple. Recently, transmission dynamic modelling of OC43 and HKU1 in the USA based on weekly laboratory testing for both viruses showed peak winter infections occurring each year for OC43 but every 2 years for HKU1. Using susceptible, exposed, infected, recovered, susceptible (SEIRS) best fit models suggested immunity to both viruses remains for 45 weeks and that immunity to OC43 provides stronger cross-immunity to HKU1 than the reverse, consistent with Dijkman *et al*.’s results [21].

## Reinfection by seasonal human coronaviruses in controlled human infection models (CHIMs)

Another way to distinguish between infection due to virus escape from neutralization, or infection in the presence of sub-protective antibody responses, is to attempt to experimentally infect adult volunteers with seasonal human coronavirus, either in the presence of their pre-existing immunity or by rechallenge with a homologous virus. Inoculation of healthy adult volunteers with the endemic coronavirus 229E led to infection in 10/15 people and clinical symptoms in 8 of those 10 infected people, even though most must have already experienced 229E infection previously in their lives. All those infected had increased antibody titres within 3 weeks of infection, which declined rapidly by 12 weeks and returned to baseline by 52 weeks. When rechallenged at 1 year, 66 % (6/9) became reinfected, but none developed clinical symptoms [22]. There are no data about the levels of virus shedding after the first or second challenge. These data were different from those of earlier studies where reinfection by a homologous coronavirus after 1 year did not occur, but reinfection with heterologous virus produced symptoms of infection. However, in the absence of sequence information about these heterologous ‘229E-like’ CoVs and given the possibility that Reed’s volunteers were more robustly infected initially, with higher antibody titres taking longer to decay, these data are not easy to interpret [23].

## Different tests to measure SARS-CoV-2 antibodies

Antibody responses to SARS-CoV-2 infection in humans and animal models have been reported in very recently published papers and non-peer reviewed preprints. These early studies suggest that the immune response to SARS-CoV-2 is similar to that for SARS-CoV and MERS-CoV. Most infected individuals (RT-PCR-positive) begin to have detectable seroconversion 10–14 days after symptom onset, but antibody levels in some mild cases can be low or undetectable. There are no systematic and well-controlled data as yet on how long the antibodies remain and what level of antibody is associated with immune protection. In comparing studies, caution should be exercised, however, because many of the studies use different assays to measure the serological response and these are not yet calibrated against each other nor have they been shown to have sufficient sensitivity and specificity to address all serological questions.

The gold standard test for antiviral antibodies is the virus neutralization test. This measures if antibodies in a serum sample can prevent susceptible cells from being infected when the antibody is mixed with a standard challenge dose of virus. However, using this test for SARS-CoV-2 requires work inside high-containment laboratories using infectious virus. A surrogate neutralization assay uses pseudotyped virus (PV) particles that bear the spike protein of the SARS-CoV-2 virus. PV neutralization assays can be performed at lower containment levels and are read out with a suitable reporter, such as luciferase, meaning that they should be scalable. Immunofluorescent (IF) tests also use virus-infected cells but detect the presence of antibodies in the sample through their reaction with viral antigens expressed in the fixed cells without assessment of the functionality of the antibodies.

Alternatively, ELISA tests and lateral flow assays (LFAs), which do not measure the function of the antibody, detect binding to a given antigen. The antigen is usually a recombinant protein such as whole spike protein, although some tests use a spike subdomain (S1) or the receptor-binding domain (RBD). It is possible that the smaller the spike fragment used, the less likely it is that antibodies in the sera raised against other endemic human coronaviruses will cross-react. However, a recent study comparing three CE-marked commercial ELISA assays and six point-of-contact (POC) tests that were available in Denmark, showed the limitations of current serological assays [24]. Thirty serum samples from severe COVID patients were assessed, along with 10 negative sera and another 71 sera from people with other viral infections to test for specificity. The Wantai SARS-CoV-2 total antibody ELISA that has spike RBD as the antigen was the most sensitive test; 100 % of day 10 samples were positive. The Euroimmun IgG test was less sensitive and only detected 78 % of the same samples. In addition, the Euroimmun IgG ELISA showed poor specificity because it detected antibodies in three sera from patients not infected by SARS-CoV-2. A similar study compared a spike trimer ELISA with nine commercial LFAs, showing that for individuals where sera was obtained after more than 31 days from symptom onset the ELISA could detect all positive samples, but the LFA tests were only consistently positive for 44 % (8/18) of these samples. Both assay types were less reliable (more false negatives) when detecting people early (<9 days) after symptom onset [25]. Similar results were obtained in a study comparing 10 LFAs and 2 ELISAs, which also highlighted the need for training and standardized LFA band intensity cutoffs for the people reading the assays [26].

Currently, the assessment of different assay formats and performance is clouded by the combination of assay sensitivity and the time taken to obtain serum sample post-symptom onset. Improvements in SARS-CoV-2 serology are still needed, but progress is rapid, as seen with the recent truly scalable commercial assays from Roche and Abbot, with reported 100 % sensitivity and 99.9 % specificity when testing over a thousand control serum samples for the Abbot assay [27]. This suggests that many types of ELISA-based tests will have better overall sensitivity and specificity performance when testing people after sufficient time has elapsed for reliable seroconversion, most likely 3–4 weeks after symptom onset. Accurate serology assays will also be essential in the testing and deployment of vaccines, either for testing at the beginning or the end of clinical trials, or through allowing serological end points as markers for other intervention strategies [28]. However, as most current vaccines and serological assays focus on the spike protein of SARS-CoV-2, human vaccinology will need to adopt serology methods from animal vaccine development, where differentiation of infected from vaccinated animal (DIVA) diagnostics are required to differentiate vaccination from infection seroconversion [29].

## Antibody responses reported in SARS-CoV-2 patients

A study of 173 people admitted to hospital in China with acute respiratory infection syndromes and/or abnormalities in chest computed tomography (CT) images [30] used 3 different assays to measure seroconversion. Similar to the Wantai commercial test above, one measured total antibody to the spike receptor-binding domain (RBD), the second measured IgM to the same spike RBD antigen and the third assay measured IgG against nucleoprotein (N). The first assay detected positive sera in 93.1 % (161/173) of patients, with a median response time of 11 days, the second measured a seroconversion rate of 82.7 % (143/173), median response time 12 days, but the response rate for IgG to the nucleoprotein was lower at 64.7 % (112/173) and took longer to appear, with a median response time of 14 days. In later samples collected 15–39 days from disease onset, the assay that measured spike RBD antibodies detected seroconversion in 100 % of patients, whereas the other assays were less sensitive (RBD IgM in 94.3 % and N IgG in 79.8 % of patients). Thus, SARS-CoV-2 seroconversion occurs on a time course that is consistent with other epidemic CoVs and antibodies to spike RBD were the most reliable for case counting in this study. At 2 weeks post-symptom onset, antibody titres were statistically higher in critical compared to non-critical patients, possibly due to different rates to a maximal antibody response or reflecting similar disease severity observations from MERS-CoV and SARS-CoV patients as described above [30]. A large-cohort serology study of 285 COVID-19-positive patients of which 262 had a record of disease symptoms, and 39 were severe infections from 3 hospitals in Hubei province, determined the antibody response to nucleoprotein and a peptide from spike protein of SARS-CoV-2. This showed that all patients seroconverted by 17–19 days after symptom onset and that severely ill patients had a significantly higher IgG titre compared to non-severe cases 7–14 days post-symptom onset, but that by 15–21 days there was no difference in the mean antibody titre between these groups. However, a considerable range of antibody titres from low to high was clearly seen in the non-severe group, while IgG titres entered a plateau within 6 days after the first positive samples [31]. Similar results continue to accumulate in other serological studies from China [32].

In a separate European collaborative study, in-house and commercial ELISAs together with a virus neutralization assay were used to measure antibodies in a total of 19 severe and mild cases. A temporal study of seroconversion in three patients showed that the patient with severe disease became antibody positive earlier than the other two patients who had mild disease; indeed, one mild patient only gave a positive serum sample using the nucleocapsid ELISA or the neutralization test and only at 28 days after symptom onset [33] . Further, in nine mild cases from early in the German outbreak, antibody responses were measured by neutralization assay and by immunofluorescence detecting IgG- and IgM-binding antibodies. There was incomplete correlation between the titres using different assays, with seroconversion occurring by day 7 in 50 % of patients and in all patients by 14 days after symptom onset. The onset of the antibody response, however, did not result in a rapid decline in virus shedding [34]. In contrast, the timing and functionality of the immune response to SARS-CoV-2 infection was considered in a detailed study of a single female patient with moderate disease in Australia. The appearance of antibody-secreting cells, T follicular helper cells and CD8-positive T cells in the blood of this patient at day 7–9 was coincident with a fall in virus titre and recovery [35]. The antibody response was also investigated in 23 patients in Hong Kong [36, 37], showing that the correlation between virus neutralization activity and IgG titres to nucleoprotein and the S1 RBD were excellent. Antibody trajectories over 20 days from this small number of severe and mild cases again demonstrated variability in individual early antibody responses, which in this study did not correlate with disease severity. A further large study of a recovered cohort of 175 patients in Shanghai measured neutralizing antibody titres by the ability of sera to block PV entry, along with other serology assays [37]. The average time for seroconversion was 10–15 days. The typical pattern observed was that patients with more severe illness showed higher neutralizing antibody (NAb) titres. Importantly, in this study around 30 % patients showed very low Nab titres, and 10 patients (6%) who were confirmed to have been infected from having an RT-PCR-positive respiratory sample did not show any antibody response at all, even at a later time point 2 weeks after hospital discharge. In the positive samples taken 2 weeks after hospital discharge there was no evidence of antibody waning. Given the variation in NAb titres, it will be important to screen convalescent plasma if it is to be used for prevention or treatment.

Considerably more well-controlled serological studies are needed, with more focus on how antibody binding assays correlate with measures of serological protection from virus infection and reinfection, such as virus neutralization assays. To test the specificity of antibody assays, sera collected from individuals in 2019 or earlier can be screened, and to test for sensitivity, sera from people in whom SARS-CoV-2 infection has been confirmed by RT-PCR can be assayed. In one such study, neutralizing antibodies measured by the PV assay were not detected in sera taken in 2019 from 100 blood donors from Scotland, nor in 500 samples from Scotland in mid-March, but were detected in 5 out of 500 Scottish sera taken in late March. An ELISA assay using S antigen detected all five positive sera and also one additional sample from the later sample set [38]. The PV neutralization assay has been used to measure potent antibodies raised in rats immunized with a potential SARS-CoV-2 vaccine based on the spike protein RBD fragment. The antibodies were as potent at inhibiting PV entry as ACE2- Ig, a decoy receptor molecule and potent SARS-CoV-2 entry inhibitor [39]. This suggests that PV neutralization will be a good correlate for protective antibodies in vaccine studies, but further studies using whole-virus neutralization will be needed to confirm this.

## Studies on SARS-COV-2 antibodies in experimental animal infections

Animal studies can provide a bridge to understanding serology and protection from infection and several species are now known to be susceptible to SARS CoV-2 infection, including non-human primates, ferrets and cats [40–42]. Infected ferrets had serum neutralizing antibodies at 12 days post-infection, but so far no re-challenge experiments have been reported [40]. Rhesus macaques are susceptible to SARS-CoV-2, where infection causes a respiratory disease lasting 8–16 days, with detectable high viral loads in the nose, throat and bronchoalveolar lavages. All animals seroconverted to the spike protein and showed neutralizing antibodies by 10 days post-infection [42]. In another study in rhesus macaques, two animals were rechallenged with virus at 28 days from the primary infection, when anti-spike antibodies were detectable and neither became infected [43]. This is unsurprising as the animals were most likely at or near the peak of their seroconversion but suggests that immediate reinfection in the face of robust neutralizing antibodies to SARS-CoV-2 is not possible.

## Conclusion

**Fig. 1.**
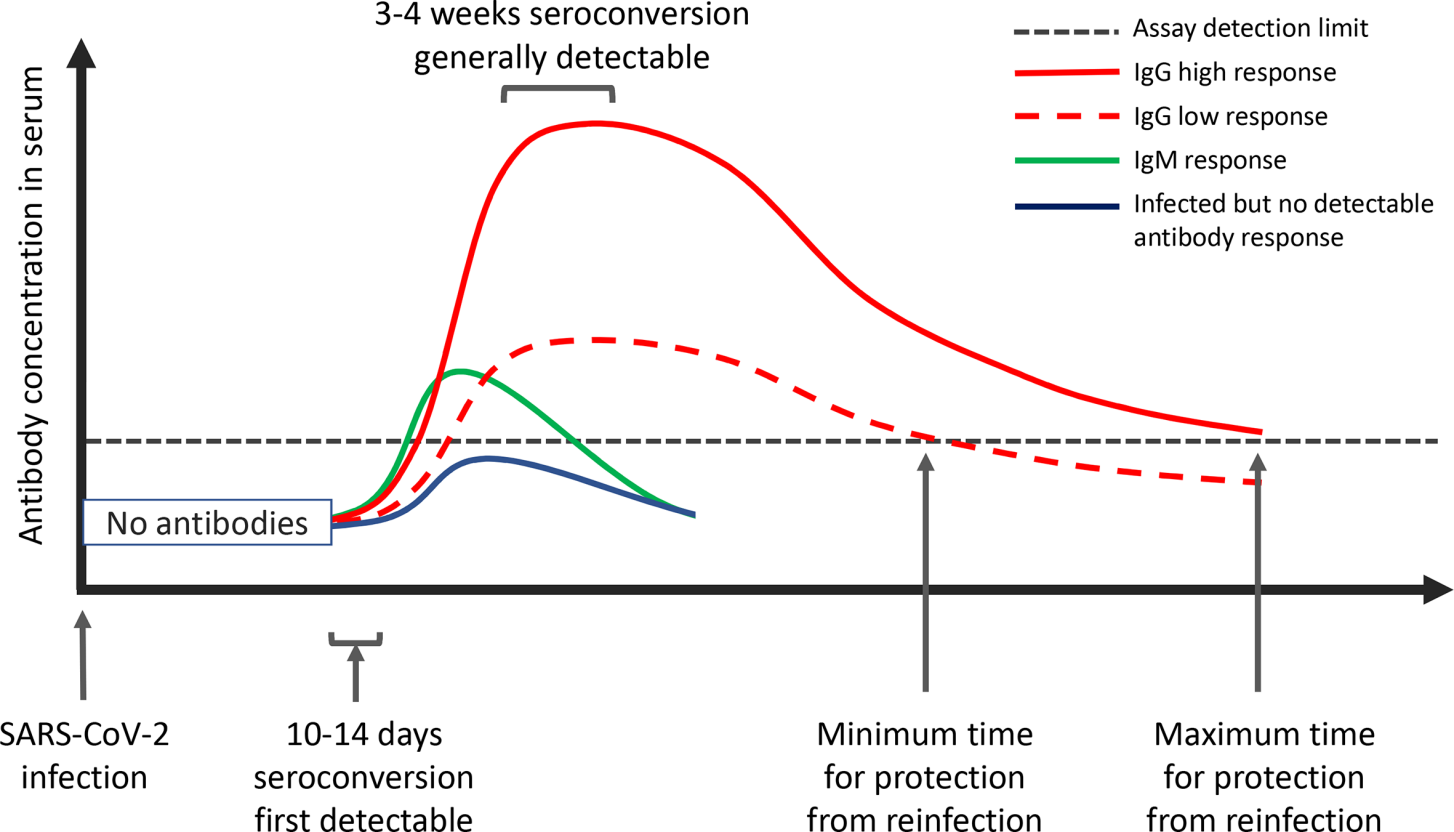
A schematic representation of the SARS-CoV-2 immune response following infection. Seroconversion occurs from approximately 10 days after symptom onset with the exact timing of IgM (green line) and IgG (red line; high titre, solid line; low titre, dashed line) appearance presently unclear, but with a suggestion that the IgM occurs at the time of, and overlapping with, the IgG response. The IgG antibody titres rise from day 10 onwards to reach a peak whose height is likely to be influenced, on a case by case basis, by disease severity and virus load. Seropositive status for those that seroconvert is detectable from 3 to 4 weeks from symptom onset. The level of antibody protection from reinfection (black dotted line), the duration of the total humoral immune response above this level, and the rate of decline from mild or severe infection induced antibodies is not known for SARS-CoV-2. Similarly, the proportion of infected individuals that do not mount a protective immune response (blue line) is not known.

It is clear that most people infected with SARS-CoV-2 display an antibody response between 10 and 14 days after infection. In some mild cases, detection of antibodies requires a long time after symptoms, and in a small number of cases, antibodies are not detected at all, at least during the time scale of the reported studies (Fig. 1). There is a paucity of information about the longevity of the antibody response to SARS-CoV-2, but it is known that antibodies to other human coronaviruses wane over time, and there are some reports of reinfection with homologous coronaviruses after as little as 80 days. Thus, reinfection of previously mild SARS-CoV-2 cases is a realistic possibility that should be considered in models of a second wave and the post-pandemic era [21]. Obtaining longitudinal serological data where both binding titres and functional neutralization titres are stratified by age groups and previous disease severity status should be undertaken as a matter of urgency. Further**,** people with low antibody titres after mild disease should be followed up for evidence of reinfection and recurrent disease by regular clinical monitoring and diagnostic virus detection by RT-PCR. If reinfection is detected, serial viral load and measures of antibody status at the time of reinfection should be established. Detailed human immunology characterization and animal studies will be necessary to determine if prior infection leads to an altered disease course if reinfection occurs. It is possible and likely that protective mechanisms through other arms of the immune response (memory and cytotoxic T cells) alter the COVID-19 disease course upon reinfection either by diminishing symptoms in the absence of protective antibodies or by enhancing infection at the nadir of the humoral immune response by sub-neutralizing antibody titres. It is also unclear if reinfections will result in onward transmission, but that cannot be excluded. Recent modelling studies, however, suggest that waning humoral immunity could have a major impact in the course of SARS-CoV-2 becoming the fifth endemic human coronavirus. Under the assumption of waning immunity across the population of the USA, similar to OC43 and HKU1, models show that if immunity is not permanent many epidemiological scenarios lead to SARS-CoV-2 becoming a seasonal human coronavirus, with either annual, biennial or sporadic patterns of epidemics over the next 5 years [21]. This means susceptible, exposed, infected, recovered, susceptible (SEIRS) models will need to replace susceptible, infected, recovered (SIR) models to inform strategies to exit from the current policies of complete transmission suppression. Serological studies will need to supply data for parameter estimates in these models as well as inform vaccine deployment to achieve maximum effects when initial supplies of vaccine will be limited. If SARS-CoV-2 immunity can be engineered to be permanent by regular vaccination, models suggest that SARS-CoV-2 infection can be dramatically reduced or possibly eliminated [21].
